# Towards Curative Cancer Immunotherapy: Overcoming Posttherapy Tumor Escape

**DOI:** 10.1155/2012/124187

**Published:** 2012-05-31

**Authors:** Gang Zhou, Hyam Levitsky

**Affiliations:** ^1^Cancer Immunotherapy Program, Cancer Center, Georgia Health Sciences University, Augusta, GA 30912, USA; ^2^Department of Medicine, School of Medicine, Georgia Health Sciences University, Augusta, GA 30912, USA; ^3^Cancer Immunology Experimental Medicine, Hoffmann-La Roche Inc., Nutley, NJ, USA; ^4^Sidney Kimmel Comprehensive Cancer Center, Johns Hopkins University School of Medicine, Baltimore, MD, USA

## Abstract

The past decade has witnessed the evolvement of cancer immunotherapy as an increasingly effective therapeutic modality, evidenced by the approval of two immune-based products by the FDA, that is, the cancer vaccine Provenge (sipuleucel-T) for prostate cancer and the antagonist antibody against cytotoxic T-lymphocyte antigen-4 (CTLA-4) ipilimumab for advanced melanoma. In addition, the clinical evaluations of a variety of promising immunotherapy drugs are well under way. Benefiting from more efficacious immunotherapeutic agents and treatment strategies, a number of recent clinical studies have achieved unprecedented therapeutic outcomes in some patients with certain types of cancers. Despite these advances, however, the efficacy of most cancer immunotherapies currently under clinical development has been modest. A recurring scenario is that therapeutic maneuvers initially led to measurable antitumor immune responses in cancer patients but ultimately failed to improve patient outcomes. It is increasingly recognized that tumor cells can antagonize therapy-induced immune attacks through a variety of counterregulation mechanisms, which represent a fundamental barrier to the success of cancer immunotherapy. Herein we summarize the findings from some recent preclinical and clinical studies, focusing on how tumor cells advance their survival and expansion by hijacking therapy-induced immune effector mechanisms that would otherwise mediate their destruction.

## 1. Introduction

Numerous studies utilizing a variety of animal models have firmly established that the host immunity fundamentally affects cancer development and progression through a process termed cancer immunoediting [1]. The immunoediting process consists of three distinct phases: elimination (host immune cells act to destroy tumor cells), equilibrium (residual tumors persist but their outgrowth is held in check by host immunity), and escape (outgrowth of tumor cells with reduced immunogenicity and/or increased capacity to attenuate or subvert host immunity). Compatible with the cancer immunoediting hypothesis, there is mounting evidence that a natural, unmanipulated host immune system can detect and respond to a developing tumor. The host-tumor interactions proceed through the three immunoediting phases either independently or in sequence, and the composite result of the process determines the outcome of tumor rejection, dormancy, or progression. Therefore, the presence of clinically apparent tumors indicates a failed attempt to control tumor progression by the host immunity due to its ineffectiveness or acquired tolerance. Thus, the goal of cancer immunotherapy is to elicit an effective antitumor immunity by engendering productive immune responses and breaking tumor-induced immune tolerance. It has been proposed that the cancer immunoediting process also occurs in humans and in therapeutic settings when established tumors are confronted by the host immunity that has been subjected to therapeutic manipulations [2]. Accordingly, the net result of immunoediting after therapy could be either cure (complete tumor eradication), or prolonged remission (persistence of dormant residual tumors), or relapse (tumor escape and progression). A multitude of cancer immunotherapy strategies have been developed with the goal to achieve the first two outcomes.

## 2. Recent Advances in Cancer Immunotherapy

A more comprehensive review on the advances in the field of cancer immunotherapy can be found elsewhere [3–5]. Here, we briefly summarize some recent progresses, with the intention to outline the therapeutic strategies and reagents that may unexpectedly elicit counterproductive effects under certain circumstances.

### 2.1. Cancer Vaccines

The premise of therapeutic cancer vaccine is that tumor-reactive T cells (including CD8+ and CD4+ T cells) can be induced and expanded in patients by exposing the host immune system to tumor-associated antigens (TAAs). Numerous vaccine approaches have been developed to deliver tumor antigens to patients, aiming to induce, activate, and amplify tumor-specific T cells. Tumor antigens can be delivered in the form of antigenic peptides, recombinant proteins, DNA or RNA constructs, recombinant microbial vectors, tumor cell lysates, and irradiated whole tumor cells. Tumor antigens are expected to be uptaken and presented by professional antigen-presenting cells (APCs), that is, dendritic cells (DCs), thereby activating tumor antigen-specific T cells. It is generally believed that the activation status of DCs critically influences the effectiveness of vaccines. In this regard, granulocyte macrophage colony-stimulating factor (GM-CSF) is widely used as a DC-activating adjuvant. Irradiated, autologous, whole tumor cells engineered to produce GM-CSF (GVAX) have been used to immunize patients with metastatic melanoma, pancreatic cancer, renal cell cancer, prostate cancer, and lung cancer [6–10]. GM-CSF-secreting allogeneic tumor vaccines have also been employed to treat multiple types of cancer [10–12]. Sipuleucel-T, the first patient-specific vaccine approved by the FDA, is formulated by incubating patient-derived peripheral mononuclear cells with a fusion protein consisting of GM-CSF and a tumor-derived differentiation antigen (prostatic acid phosphatase) [13]. Besides GM-CSF, other major vaccine adjuvants include bacilli Calmette-Guerin (BCG) and toll-like receptor (TLR) agonists, for example, poly-ICLC for TLR3, LPS, and synthetic TLR4 agonists, imiquimod for TLR7, and CpG for TLR9.

### 2.2. Adoptive Cell Therapy (ACT)

ACT is a form of immunotherapy which involves the transfusion of large numbers of autologous or allogeneic, tumor-reactive lymphocytes to tumor-bearing hosts. The source of autologous tumor-reactive lymphocytes can come from lymphocytes infiltrating the tumor (TIL) or bone marrow (MIL), or peripheral blood mononuclear cells (PBMC). The specificity of the lymphocytes used for transfer could be either polyclonal (reactive to multiple undefined tumor antigens), or monoclonal (specific for a single defined tumor antigen). Unfractionated polyclonal TILs, after *ex vivo* expansion, have been used to treat patients with metastatic melanoma, in conjunction with systemic recombinant IL2 [14, 15]. ACT using tumor-specific T-cell clones relies on the ability to isolate and activate antigen-specific T cells from patients' specimens and then clonally expand these cells by reiterative antigenic stimulation. Although this strategy has generated encouraging results in melanoma clinical trials, its broad application has been hindered by the need of a series of individualized and cumbersome procedures required to obtain sufficient numbers of tumor-specific T cells. These limitations can be circumvented by the transfer of PBMC-derived lymphocytes that have been transduced to express T-cell receptors (TCRs) with the desired antigen specificity. ACT using lymphocytes bearing genetically engineered TCRs exhibited therapeutic efficacy in patients with metastatic melanoma as well as other forms of cancer [16]. A variation of this TCR engineering strategy is the generation of chimeric antigen receptors (CARs) which combine the antigen-binding properties of a monoclonal antibody (extracellular domain) and intracellular T-cell signal transduction domain consisting of CD3- zeta chain in conjunction with costimulatory endodomains such as CD28, OX40, or 4-1BB. Recent clinical trials conducted by different groups using lymphocytes bearing CD19-specific CARs reported encouraging clinical responses in patients with B-cell malignancy [17–19]. Of note, a preparative chemotherapy regimen is routinely used prior to ACT to induce transient lymphodepletion which facilitates the engraftment, expansion, and survival of the infused lymphocytes [20].

### 2.3. Therapeutic Monoclonal Antibodies and Immune-Modulating Antibodies

Monoclonal antibodies (mAbs) have proven to be valuable therapeutic agents for cancer treatment resulting in clinical responses and survival benefits in some patients. Currently, there are eight clinically approved therapeutic mAbs targeting five tumor-associated proteins, including CD20 (rituximab, Ibritumomab tiuxetan, and tositumomab), CD33 (gemtuzumab), CD52 (alemtuzumab), HER2/neu (trastuzumab), and EGFR (cetuximab, panitumumab). These mAbs can directly target malignant cells and exert antitumor effects by antagonizing oncogenic pathways and opsonizing tumor cells to trigger antibody-dependent cellular cytotoxicity or phagocytosis [21]. It has also been suggested that tumor-targeting mAbs may enhance tumor antigen uptake and presentation, thereby activating antitumor T-cell responses [22, 23]. In addition, mAb bevacizumab blocks tumor angiogenesis by inhibiting vascular endothelial growth factor-A (VEGF-A) expressed by host cells.

It is known that the host immune system has evolved to control the balance between immune activation and tolerance with a set of delicate intrinsic mechanisms involving the functions of costimulatory or coinhibitory molecules. These immunomodulating mechanisms, which normally enable the host immunity to respond to invading pathogens while maintaining homeostasis, often become dysregulated in the presence of active malignancy. In moving beyond antibodies that directly target and kill tumor cells, a different class of mAb has emerged as important and potent modulators for productive immune responses. These mAbs target the costimulatory or coinhibitory receptors expressed on activated T cells, and their corresponding ligands on APCs or tumor cells. The rationale is to accentuate the stimulatory signals with agonist mAbs or disrupt the inhibitory signals with antagonist mAbs (checkpoint blockade) [24, 25]. The prototypical immunomodulating antibody is ipilimumab directed against CTLA-4, a checkpoint molecule that negatively regulates T-cell activation and function. Administration of ipilimumab, either alone or in combination with a peptide vaccine or chemotherapy, demonstrated long-term survival benefits in patients with metastatic melanoma in randomized phase III clinical trials [26, 27]. Programmed death 1 (PD-1) is another inhibitory receptor expressed on activated T cells. PD-1 interacts with its two ligands PD-L1 and PD-L2. PD-L1 is broadly expressed on APCs, nonimmune tissues, and tumor cells, and its expression correlates with an unfavorable prognosis in multiple types of cancer [28]. Sustained expression of PD-1 on tumor-reactive T cells is associated with a functionally exhausted phenotype [29–31]. Humanized anti-PD-1 and anti-PD-L1 antibodies have been developed and are currently under clinical evaluations. Phase I trials conducted in patients with several types of solid tumors demonstrated that PD-1 blockade was well tolerated and can achieve objective responses in some patients [32]. Besides CTLA-4 and PD-1, T-cell immunoglobulin mucin-3 (Tim-3) and lymphocyte-activation gene-3 (LAG-3) appear to be potential targets for antibody blockade, based on accumulating evidence that both LAG-3 and Tim-3 synergize with PD-1 to attenuate antitumor T-cell responses [33–35].

Parallel to the development of antagonist antibodies against checkpoint proteins, monoclonal antibodies acting as agonists of stimulatory receptors have also been generated for the purpose of augmenting antitumor immune responses. These antibodies mainly target a group of TNF family costimulatory receptors, including CD40, CD134 (OX40), CD137 (4-1BB), and glucocorticoid-induced tumor necrosis factor receptor (GITR). Among these antibodies, agonist anti-CD40 Abs have been extensively studied and exhibited clinical activities in a range of tumor types [36–38].

### 2.4. Combination Therapy

The microenvironment of a growing tumor is rendered profoundly immunosuppressive by a variety of mechanisms [39]. The well-characterized mechanisms include immunosuppression mediated by regulatory T cells (Tregs), myeloid-derived suppressor cells (MDSCs), metabolizing enzyme indoleamine 2,3-dioxygenase (IDO), inhibitory molecule PD-L1, and immunosuppressive soluble factors (such as IL-10, TGF*β*, prostaglandin E2, and VEGF). CD4^+^Foxp3^+^ Treg cells can negatively modulate DC functions and suppress the effector activities of helper CD4+ T cells (Th), cytotoxic CD8+ T cells (CTL), and natural killer cells (NK). The mechanisms of Treg-mediated suppression are not entirely clear, but may involve inhibitory surface molecules CTLA-4 and PD-1, immunosuppressive soluble factors TGF*β*, IL-10, and IL-35, and cytolytic molecules granzyme B and perforin [40]. MDSC can suppress immune responses through the activities of arginase 1, inducible nitric oxide synthase (iNOS), nitric oxide (NO), and reactive oxygen species (ROS) [41]. IDO and the related enzyme Tryptophan-2,3-dioxygenase (TDO) catalyze the degradation of the essential amino acids tryptophan (Trp) into kynurenine (Kyn), resulting in immune tolerance by reducing the local concentration of Trp required by T cells, and by the direct immune-inhibiting effects of Kyn [42]. In addition, Kyn has recently been identified as a natural ligand for the aryl hydrocarbon receptor (AHR) [43], which is involved in tumorigenesis, inflammation, and Treg development [44, 45]. It has been shown that IDO-expressing DCs can activate Tregs, which in turn mediate suppression through the PD-1/PD-L1 pathway [46]. These immunosuppressive mechanisms may operate simultaneously or sequentially, forming a mutually compensatory and self-reinforcing tolerogenic network. Conceivably, any form of immunotherapy has to overcome these hurdles to be effective. At present, the consensus in the field is that a combinatorial strategy has to be taken to target multiple immune pathways to attain durable antitumor effects.

Various forms of immunotherapy have been used in combination with conventional chemotherapy. Notably, most adoptive cell therapy protocols contain a lymphodepleting chemotherapy regimen in which the alkylating agent cyclophosphamide (CTX) is a major component. The immunoenhancing effects of CTX include “creating space” (i.e., providing access to limiting concentrations of cytokines and survival factors), depleting/inactivating Tregs [47], inducing the release of multiple growth factors and proinflammatory cytokines/chemokines [48, 49], all of which promote donor cell activation, expansion, survival, and memory formation [50, 51]. In terms of cancer vaccines, it has been well documented that chemotherapy can enhance the efficacy of GM-CSF whole-cell vaccines in both humans and mice [52, 53]. For immunomodulating antibodies, the combined use of chemotherapy with anti-CD40 agonist mAb [38], or anti-CTLA-4 antagonist mAb ipilimumab [27], both generated objective responses in patients.

A large body of preclinical work has shown enhanced therapeutic efficacy by combining different forms of immunotherapy. Administration of adjuvant IL-7 can enhance vaccine-induced antitumor immune responses by fostering a proinflammatory milieu and antagonizing Treg suppression and TGF*β* signaling [54]. IFN*α* has been shown to enhance GM-CSF vaccine and peptide vaccine [55, 56], likely due to its immunostimulatory effects on DCs and T cells but inhibitory effects on Tregs [57, 58]. Conjugation of IFN*α* with a therapeutic antibody targeting CD20 seemed to increase tumor cytotoxicity while reducing the side effects associated with IFN*α* [59, 60]. In a mouse model of metastatic renal cancer, the use of anti-CD40 agonist mAb in combination with IL-2 or IL-15 can mediate tumor regression by inducing an inflammatory milieu that inhibited intratumoral Treg and MDSC activities [61]. The combined use of antibody blockade targeting different checkpoint molecules, such as anti-CTLA-4 and anti-PD-1 mAbs, anti-PD-1 mAbs, and anti-Tim-3 or anti-Lag-3, exhibited additive or synergistic effects in preclinical models [33, 35, 62], prompting the design of parallel clinical studies.

In summary, the growing availability of novel immunotherapeutic reagents offers immense options for combination therapy. These and many other potentially synergistic treatment combinations are areas of active investigation. Importantly, whereas combination therapies were historically developed empirically, with only complementary dose limiting toxicities as a driving principle, mechanistic studies of discrete elements of the host immune response provide a rationale for specific combinations, the choice of which may ultimately be tailored to individual patients (“personalized health care”).

## 3. Immunological Features Associated with Effective Therapies

The onset, magnitude, and duration of the elicited immune responses vary dramatically from different forms of immunotherapies and vary from different individuals undergoing the same therapy. Currently, there remains a knowledge gap between the observed variations in therapy-elicited immune responses and the ultimate treatment outcomes, which may range from durable remission, disease stabilization, to initial remission followed by relapse, and exacerbated tumor progression. Here, we attempt to summarize the common features of the immune responses elicited by effective therapies—those that have led to beneficial outcomes in clinical studies or demonstrated efficacy in preclinical models. These features may shed light on the mechanisms of tumor immune escape in some cases, whereby an initial robust antitumor immunity elicited by therapy is subsequently rendered tolerant and unproductive.

### 3.1. Therapy-Induced Acute Inflammation

It has become increasingly clear that inflammatory responses play critical roles at different stages of tumor development, including initiation, progression, and metastasis [63]. These inflammatory responses are usually chronic in nature and inherent to many types of cancer, especially solid malignancies. Nonetheless, inflammation can also be induced by various cancer therapies, although it remains controversial whether therapy-induced inflammation is beneficial or detrimental to the hosts [64, 65].

It is not unexpected that many cancer immunotherapies, especially those involving chemotherapy, cause inflammation in treated hosts. Chemotherapeutic agents such as doxorubicin and oxaliplatin can cause massive tumor cell death and tissue damages, releasing various danger signals, such as high-mobility group protein B1 (HMGB1), calreticulin (CRT), and adenosine triphosphate (ATP) [66–68]. These danger/stress signals activate the innate immune system, resulting in rapid production of proinflammatory cytokines such as IL-1*β* and IL-17, leading to the activation of the adaptive immune system [67, 69]. Another well-studied chemotherapeutic agent is cyclophosphamide (CTX), which has been shown to rapidly induce the release of growth factors (including IL-2, IL-7, IL-15, and GM-CSF), proinflammatory cytokines (including IFN*γ*, IFN*α*/*β*, IL-1*β*, IL-6, and IL-17), and chemokines in treated tumor-bearing hosts [48, 49].

In some cases, inflammation can be induced by tumor-reactive T cells. This is exemplified by a recent clinical trial in which a patient with refractory chronic lymphocytic leukemia (CLL) received infusion of CD19-specific CAR-modified autologous T cells following chemotherapy preconditioning with CTX [18]. Complete remission was achieved 3 weeks after treatment, and the clinical response was accompanied by a delayed, temporal increase of inflammatory cytokines IFN*γ* and IL-6, and IFN*γ*-responsive chemokines CXCL-9 and CXCL-10, in blood and bone marrow. Since chemotherapy, like CTX used in this trial, usually induces a rapid and transient surge of inflammatory cytokines/chemokines that resolve in days [48], thus the delayed (15–30 days after T-cell infusion) emergence of inflammatory cytokines was likely the consequence of T-cell-mediated antitumor responses.

Moreover, immunotherapies without the involvement of chemotherapy can also induce inflammation. In an animal model in which vaccination was administered in conjunction with adjuvant IL-7, improved antitumor responses and survival were associated with increased serum concentration of IL-6, IL1*α*, IL-1*β*, IL-12, IL-17, TNF*α*, and chemokines CCL-5 and CCL-3 [54]. In a mouse model of renal cancer, the efficacy of IL-2/anti-CD40 agonist antibody was associated with conversion of the immunosuppressive tumor milieu to an immunogenic milieu which was rich of inflammatory chemokines including CXCL-9, CXCL-10, CCL-5, and CCL-3 [61]. Although the underlying mechanism of therapy-induced inflammation has not been defined, some recent studies suggest that tumor-reactive CD4+ T cells play an important role in initiating and modifying the inflammatory milieu [70–72].

Taken together, effective cancer therapies tend to generate an acute-type inflammation, which is typically associated with increased levels of IL-1, IL-6, IL-17, IFN*γ*, IFN*α*/*β*, and several IFN*γ*-responsive chemokines.

### 3.2. Mitigation of Local Immunosuppression

Since the discovery of the immunosuppressive properties of Treg cells, depletion or inactivation of Tregs has become an important component of many immunotherapeutic strategies. Low-dose CTX can effectively deplete Tregs and restore robust antitumor immune responses [47, 73]. Imatinib (Gleevec), a tyrosine kinase inhibitor used as targeted therapy for several types of cancers, was recently shown to promote Treg apoptosis [74]. Some immunotherapeutic agents blunt Treg-mediated suppression by mechanisms other than systemic depletion of Treg cells. Anti-CTLA-4 antagonist mAb (ipilimumab) does not reduce Treg cell numbers but instead preferentially exclude Tregs from the tumor lesion, so as to increase the intratumoral *T*_effector_/*T*_reg_ ratio and improve therapeutic efficacy [75, 76]. Anti-OX-40 agonist mAb has been shown to promote tumor rejection by inactivating Tregs [77–79]. Interestingly, coadministration of CTX and anti-OX-40 agonist mAb in a mouse melanoma model led to profound intratumoral Treg depletion accompanied by an influx of effector T cells [80], suggesting that combined use of multiple Treg-targeting agents may be more effective.

## 4. Counterregulation Mechanisms That May Lead to Posttherapy Tumor Escape

It is not uncommon both in clinical studies and animal models that some cancer immunotherapies can induce measurable immune responses, but these responses did not translate into durable beneficial outcomes, suggesting the occurrence of tumor immune escape. Many factors may contribute to tumor escape under the immune pressure imposed by therapies, including immunogenicity alterations (downregulation of MHC expression, loss of antigen) in tumor cells in response to therapy, amplification of immunosuppressive cells (Tregs, MDSCs), and induction of immune checkpoint molecules in effector T cells (CTLA-4, PD-1). There is emerging evidence that certain components of the therapy regimen, and/or some immune products induced by therapy, may induce or exacerbate some of these tumor escape pathways. In this section, we discuss the possible mechanisms by which tumors antagonize and subvert robust immune responses in the posttherapy setting, thereby promoting tumor escape.

### 4.1. Treg Expansion or Repopulation after Therapy

Many cancer immunotherapies use vaccines to induce and amplify tumor-specific T cells. However, we initially reported that a vaccinia virus-based therapeutic vaccine indiscriminately expanded both CD4+ effector T cells and Tregs in tumor-bearing hosts, resulting compromised antitumor immune responses [81, 82]. This observation was further confirmed in various animal tumor models and clinical studies. In a spontaneous murine mammary tumor model, repeated IL-12/GM-CSF therapy led to a progressive increase of tumor-infiltrating Treg cells that impeded long-term antitumor effects [83]. In a melanoma animal model, LaCelle et al. reported that multiple rounds of GM-CSF whole-cell vaccinations during immune reconstitution of the lymphopenic hosts augmented the number of Treg cells and were associated with diminished antitumor potency of the T-cell repertoire [84]. In patients with HPV16-induced vulvar intraepithelial neoplasia, therapeutic vaccination with HPV16 E6/E7 synthetic long peptides led to increased frequencies of HPV16-specific Treg cells in a subset of patients, among which clinical responses were absent [85].

IL-2, a T-cell growth factor, has been approved by the FDA for the treatment of patients with metastatic melanoma and renal cell carcinoma. However, only 15% to 20% of treated patients experienced a clinical response, with 7% complete long-term responders [86]. The limited efficacy is likely in part due to the fact that IL-2 can significantly increase the frequency of functional Treg cells [86–88]. Indeed, daily injection of low dose of IL-2 has recently been reported to be successful in treating the immunopathology associated with *chronic *graft versus host disease in association with amplification of Treg numbers in allogeneic bone marrow transplant recipients [89].

TLR ligands have been frequently used as immune adjuvants to enhance antitumor immunity and are thought to be able to break immune tolerance by directly or indirectly inhibiting Treg responses [90–92]. However, it has been reported that some TLR agonists, including TLR4 ligand LPS and TLR5 ligand flagellin, can induce the proliferation and enhance the suppressive function of Treg cells [93, 94]. It is increasingly recognized that TLR agonists can generate suppressive as well as inflammatory responses in innate immune cells and can promote the induction of regulatory as well as effector T cells [95].

Currently available approaches that seek to abrogate Treg-mediated suppression, including cyclophosphamide, anti-OX-40 agonist mAb, and anti-CTLA-4 antagonist mAb, all seem to have limitations. Depletion of Treg cells by cyclophosphamide is transient and followed by rapid Treg repopulation, thus repeated administration of this drug may be required to stem Treg recovery [96, 97]. Recent studies suggest that anti-OX-40 agonist mAb may have dual functions on Tregs depending on environmental cues such as the local cytokine milieu. One study reported that OX40 signaling regulated Treg responsiveness to IL-2 and was required for sustaining Treg competitive fitness *in vivo* during repopulation of lymphopenic hosts [98]. Another report showed that anti-OX-40 agonist mAb blocked TGF-*β*1-mediated Treg conversion of activated T cells through enhanced production of Th1 or Th2 cytokines but promoted Treg proliferation and survival when IFN*γ* or IL-4 was absent in the local environment [99]. Anti-CTLA-4 antagonist mAb (ipilimumab), though proven to be effective in reducing the presence of Tregs within tumor, actually expands the overall number of Treg cells [75, 76, 80]. These features present the potential risk that Tregs may reestablish and repopulate in residual tumors and hence compromise long-term therapeutic effects.

Some therapeutic maneuvers, such as total body irradiation (TBI) followed by bone marrow transplantation in conjunction with infusion of immune lymphocytes, have also been shown to preferentially expand T-effector cells over Treg cells in a limited time window [100, 101]. However, host-derived radioresistant residual Tregs can rebound to occupy a niche in lymphopenic transplantation recipients [102], and functional Tregs de novo induced from the donor lymphocytes can repopulate in the hosts to reestablish immune tolerance [103, 104]. 

### 4.2. Expansion of MDSC

GM-CSF cancer vaccines can potently stimulate antitumor immune responses, in part, by causing the growth and differentiation of DCs [105]. However, it has been documented that chronic production or high-doses of GM-CSF can adversely affect antitumor immune responses by recruiting and expanding immunosuppressive MDSCs in animal tumor models [106–108]. Similar adverse effects of GM-CSF-based vaccines have been reported in some clinical studies [109–111].

In addition to GM-CSF, IL-1*β*, IL-6, and IL-17, inflammatory cytokines frequently induced after therapies can also drive MDSC expansion in tumor [112–114]. In fact, many inflammatory cytokines have dual immunoregulatory activities, which could either enhance or attenuate tumor immunity. For example, on one hand, IL-1*β* can promote helper T-cell differentiation and inhibit Treg suppression [115–117]. On the other hand, IL-1*β* can promote tumor angiogenesis and metastasis [118, 119]. Thus, a balanced inflammatory milieu after therapy may be critical for durable antitumor effects [120]. In the case of IL-1*β*, unbalanced levels of IL-1*β*, either too much (MDSCs preferentially expand) or too little (Tregs become prevalent), all lead to tumor progression [114].

### 4.3. Survival and Proliferation of Residual Tumors

The use of chemotherapeutic agents has become an integral component of many cancer immunotherapies. In addition to debulking of the tumor mass, some anticancer drugs benefit cancer therapy by potentiating host antitumor immunity [121, 122]. However, chemotherapy almost invariably has certain side effects. While most of the side effects are associated with the global cytotoxicity of anticancer drugs, there is emerging evidence that some agents may even exert “opposite effects” that can enhance the malignancy of the treated cancers. For example, it has been shown previously that under certain experimental settings, cyclophosphamide treatment may render mice more prone to tumor metastasis by facilitating tumor cell intravascular proliferation, extravasation, and colony formation [123]. A recent study showed that chemotherapy with cisplatin or paclitaxel can induce VEGFR-1 expression on endothelial cells, creating an environment favorable to tumor cell retention and metastasis [124]. A different study reported that conditioned medium from BMDCs and plasma from paclitaxel-treated mice can promote metastatic properties in tumor cells *in vitro* by inducing matrix metalloproteinase-9 (MMP9), and paclitaxel treatment of mice with Lewis lung carcinoma led to accelerated MMP9-dependent metastases [125]. These findings suggest that some chemotherapeutic agents can induce a cascade of host events that may potentially support the growth and spread of residual tumors. Furthermore, some inflammatory cytokines induced after chemotherapy may contribute to tumor relapse and chemoresistance [126]. In a mouse model of Burkitt's lymphoma, Gilbert and Hemann showed that IL-6 was released in the thymus in response to doxorubicin treatment, creating a “chemoresistant niche” that promotes the survival of a minimal residual tumor burden and serves as a reservoir for eventual tumor relapse [127]. Taken together, these studies illustrate that the prosurvival and prometastasis effects of some chemotherapeutic agents may confound the treatment outcomes of some cancer immunotherapies.

### 4.4. Upregulation of Checkpoint Molecules

It is well known that PD-L1 expression can be upregulated by both type I (IFN*α*/*β*) and type II (IFN*γ*) interferons, which are often induced after bacteria or viral infections, serving as a hard-wired counterregulation mechanism to avoid excessive immune responses [28, 128]. However, this mechanism also can be employed by residual tumors to counteract antitumor immunity elicited by therapy, which is often associated with the production of interferons. In addition to regulating PD-L1 expression, a recent study reported that IFN*α* can also augment PD-1 expression on antigen-stimulated T cells, rendering these T cells susceptible to PD-L1-mediated suppression [129]. Besides interferons, several common gamma-chain cytokines IL-2, IL-7, and IL-15, which are often elevated after chemotherapy, have been found to upregulate PD-1 as well as PD-L1 on T cells [130], suggesting similar mode of immune regulation.

It has been shown that PD-L1 plays an essential role in the development, maintenance, and function of Treg cells [131, 132]. Therefore, one of the anticipated benefits of PD-L1 blockade is to mitigate Treg-mediated suppression. However, a recent study presented evidence that PD-L1 blockade can expand ICOS^+^Foxp3^+^ CD4^+^ regulatory T cells, which act to inhibit the optimal functions of CTLs [133]. This result is consistent with the notion that PD-L1 may negatively regulate Treg cells under certain circumstances, as in the case of chronic infection by hepatitis C virus [134]. These data provide a cautionary note for the possible opposing effects of PD-L1 blockade on tumor immunity.

### 4.5. Induction of IDO

Many immune adjuvants used in cancer immunotherapies, including lipopolysaccharide (TLR4 ligands), resiquimod (TLR7/8 ligands), CpG (TLR9 ligands), and anti-CD40 antibody, can induce IDO expression [135]. In addition, proinflammatory cytokines IFN*γ* and IFN*α*/*β* are potent IDO inducers [136]. Given IDO's broad activities in mediating direct T-cell suppression and Treg activation [46], posttherapy IDO induction represents a constant threat to long-term therapeutic efficacy. Thus, durable tumor remission may not be achievable unless induced IDO activity is blocked concomitantly. Supporting this notion, it has been shown in various model systems that inhibition of IDO by a clinically applicable inhibitor 1-methyl-tryptophan (1MT) can markedly improve the efficacy of a wide range of cancer therapies, including cytotoxic chemotherapy [137], IL-12/GM-CSF therapy [138], and targeted therapy [74].

## 5. Overcoming Tumor Escape with Combinatorial Treatment Strategy

The immune-tolerizing mechanisms discussed in the preceding section may also operate in tumor-bearing hosts prior to therapy. These mechanisms are subject to disruption by cancer immunotherapy, but under certain conditions, they can recover and reestablish immune tolerance to residual tumor cells. The reemergence of a tolerogenic mechanism can be driven by certain components of the therapy regimen, for instance, Treg expansion by IL-2 immunotherapy; alternatively, some therapy-induced immune mediators can counterregulate antitumor immune responses, for example, type I IFN can induce PD-1 in activated T cells and upregulate PD-L1 in tumor cells, and IL-1*β* and GM-CSF can recruit and activate MDSCs. It is unlikely that these tolerogenic mechanisms act in isolation; rather, they may be interactive and mutually compensatory. Therefore, an effective cancer immunotherapy requires a combinatorial strategy to overcome tumor immune escape. With increased knowledge of tumor escape mechanism at work, rational combination of multiple targeted treatment approaches has shown tremendous potential in achieving curative outcomes in preclinical models. In a mouse renal cell carcinoma (RENCA) tumor model, Webster et al. showed that Tregs and PD-L1 collaborated to impair vaccine-induced recall response of tumor-specific memory T cells; correspondingly, treatment with tumor cell vaccines in combination with PD-L1 blockade and CD4+ T-cell depletion (triple therapy treatment) resulted in complete regression of large established tumors and raised durable protective immunity [139]. A recent study by Khleif's group reported that the combination of a tumor vaccine, PD-1 blockade, and CTX led to complete regression of established TC-1 tumors and improved survival benefits in a significant percentage of treated animals [140]. In this study, the combined use of PD-1 blockade and low-dose CTX markedly increased the number of vaccine-induced tumor-infiltrating CTLs while sustainably reducing systemic and local Tregs. In a mouse melanoma model, complete regression of advanced primary melanomas in the skin and metastases in the lung was achieved by a protocol which consisted of timed treatments of CTX preconditioning, adoptive transfer of tumor-specific CD8+ T cells, viral vector-based vaccinations, and administration of CpG-containing adjuvants [141]. These results provide compelling rationales for applying similar strategies in future clinical trials.

## 6. Conclusions

Current cancer immunotherapies rarely result in immediate and complete tumor eradication, and more often the residual tumors persist and likely form equilibrium with the host immunity, with the end result being either tumor recurrence or sustained remission. It is important to appreciate that during this process, components of the therapy regimens, such as various chemotherapeutic agents and immune adjuvants, and elements of therapy-induced responses, such as proinflammatory cytokines, can shift the balance one way or the other. The key challenge relies on synchronizing the immune-enhancing effects of all relevant factors while minimizing their collateral counterproductive effects. Our improved knowledge of the relations of tumor immunity and tumor counterregulation should help the design of more efficacious cancer immunotherapy strategies that lead to a curative outcome.
